# Virtual reality suturing task as an objective test for robotic experience assessment

**DOI:** 10.1186/s12894-015-0051-4

**Published:** 2015-07-03

**Authors:** Michael A. Liss, Christopher J. Kane, Tony Chen, Joel Baumgartner, Ithaar H. Derweesh

**Affiliations:** Department of Urology, UC San Diego Health System, San Diego, CA USA; University of California, San Diego School of Medicine, La Jolla, CA USA; Department of Surgery, UC San Diego Health System, San Diego, CA USA; UC San Diego Moores Cancer Center, 3855 Health Sciences Drive #0987, La Jolla, CA 92093-0987 USA

**Keywords:** Education, Robotics, Simulation, Virtual reality

## Abstract

**Background:**

We performed a pilot study using a single virtual-simulation suturing module as an objective measurement to determine functional use of the robotic system. This study will assist in designing a study for an objective, adjunctive test for use by a surgical proctor.

**Methods:**

After IRB approval, subjects were recruited at a robotic renal surgery course to perform two attempts of the “Tubes” module without warm-up using the Da Vinci® Surgical Skills Simulator™. The overall MScore (%) from the simulator was compared among various skill levels to provide construct validity. Correlation with MScore and number of robotic cases was performed and pre-determined skill groups were tested. Nine metrics that make up the overall score were also tested via paired *t* test and subsequent logistic regression to determine which skills differed among experienced and novice robotic surgeons.

**Results:**

We enrolled 38 subjects with experience ranging from 0- < 200 robotic cases. Median time to complete both tasks was less than 10 min. The MScore on the first attempt was correlated to the number of previous robotic cases (R^2^ = 0.465; p = 0.003). MScore was different between novice and more experienced robotic surgeons on the first (44.7 vs. 63.9; p = 0.005) and second attempt (56.0 vs. 69.9; p = 0.037).

**Conclusion:**

A single virtual simulator exercise can provide objective information in determining proficient use of the robotic surgical system.

## Background

Surgical training has met immense challenges from the rapid growth of minimally invasive surgery (MIS) across all surgical specialties, compounded by limitations in training hours [1, 2]. In particular, robotic surgery has expanding indications in many surgical specialties [3]. As with diffusion of any new technology, early adoption of robotic surgery was associated with adverse patient outcomes [4, 5]. Robotic simulation may improve the learning curve and may also improve the operative characteristics of surgeons with simulation training [6–9].

Robotic surgery encompasses new challenges in assessing skill, proctoring, and credentialing of surgeons [10]. Proctoring is an essential patient safety component of surgeon privileging for specific operations, including the use of new technology. However, the proctor has limited guidelines, assessment parameters, or objective tools to assess other surgeons and their comfort level with new technology [11].

Objective testing of the adequate use of a robotic surgical system may provide added information for institutions and proctors regarding specific surgeons’ comfort level and competency with the robotic equipment prior to live patient operative experience. We investigate whether the overall score calculated from one advanced module (“Tubes”) on the Da Vinci® Surgical Skills Simulator™ (DVSSS, Intuitive Surgical, Inc., Sunnyvale, CA, USA) is associated with a number of previous robotic cases and assumed comfort with using the robotic system safely.

## Methods

### Participants and setting

University of California San Diego Institutional Review Board reviewed and approved the ethical conduct of the study (IRB#: 130298). After IRB approval and informed consent, participants enrolled in an American Urological Association-sponsored “Hands on Robotic Renal Surgery” course on May 3, 2013 were asked to perform the “Tubes” training module on the DVSSS twice at one sitting. Each participant was given a number for confidentiality in analysis and was asked to record the number of previous robotic cases performed as primary surgeon. This identification number was entered into the simulator as their user name. After watching the instructional video at the beginning of the module, the participant was not given any other direction. The results from the individual components and the overall score were recorded within the software and retrieved after all participants completed the task.

### Simulator

**Fig. 1:**
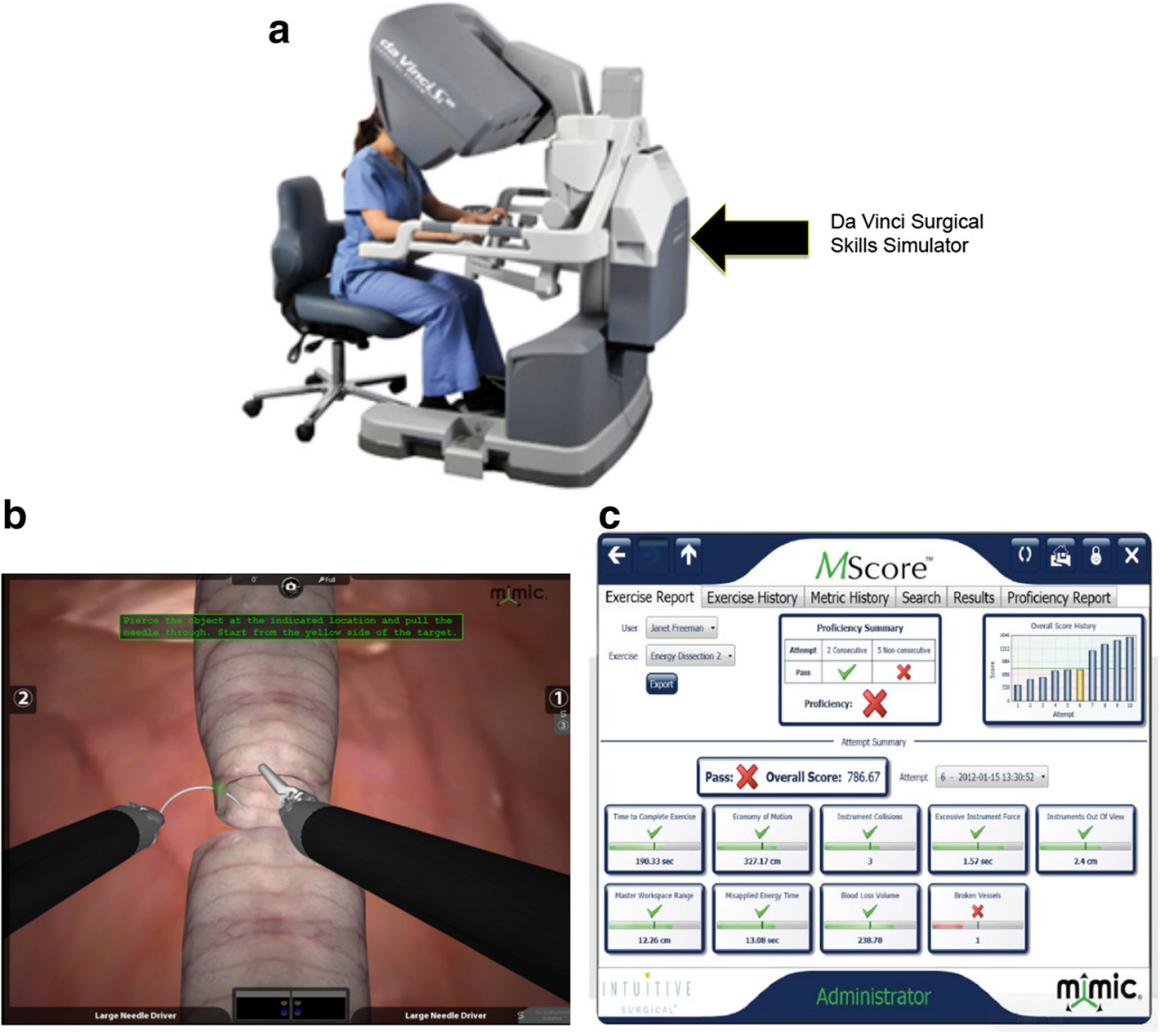
**a**: Da Vinci^®^ Surgical Skills Simulator™. **b**: Tubes module. **c**: MScore evaluation score sheet

We used the DVSSS, which incorporates the Mimic software program (Mimic Technologies, Inc. Seattle, WA, USA) to provide an MScore™ developed from individual skill metrics (Table 1, Fig. 1a and b). The MScore™ and metric percentages were developed from the mean and standard deviation of 100 robotic surgeons who have completed at least 75 robotic cases (similar to the Fundamentals of Laparoscopic Surgery FLS™ protocol) to facilitate credentialing and privileging (http://www.mimicsimulation.com/products/dv-trainer/mscore-evaluation). The DVSSS uses the actual Da Vinci Si® surgical robotic console (Intuitive Surgical, Inc., Sunnyvale, CA, USA) with the video cable to the simulation pack that fits on the posterior aspect of the console (Fig. 1a). The “Tubes” module is a virtual simulation of a suturing task mimicking an anastomosis of two tubular structures and has been validated in previous studies [12]. The simulator and software have been evaluated favorably for face, content, construct, and concurrent validity, though were found to be limited on predictive validity [6, 13–15].

**Table 1:** Metric definitions

Metric		Definitions
Overall score	-	The weighted average of metric scores
Economy of motion	1	Total distance (measured in centimeters) traveled by all instruments
Time to complete	2	Total time (measured in seconds) the user spends on the exercise
Instrument collisions	3	Total number of instrument-on-instrument collisions exceeding a minimum force threshold
Master workspace range	4	Diameter (measured in centimeters) of user’s working volume on master grips
Critical errors	5	Number of metrics whose % score is zero
Instruments out of view	6	Total distance (measured in centimeters) traveled by instruments outside the user’s field of view
Excessive force	7	Total time (measured in seconds) an excessive instrument force is applied above a prescribed threshold
Missed targets	8	Number of missed targets
Drops	9	Number of times an object or objects are dropped in an inappropriate region of the scene

### Study design and data collection

We performed a construct validity observational study of urologic surgeons and residents at the AUA course described above. The one-day course consisted of didactic learning regarding technique, simulation of robotic tasks, and hands-on robotic porcine laboratory experience. Data was collected from the DVSS in Microsoft Excel format including all metrics for the first and second attempt at the Tubes module. The database was expanded to include the number of previous robotic cases. We categorized participants into two groups. The first group included novice robotic surgeons (0–10 cases) and intermediate/experienced robotic surgeons (>10 cases) based on previous studies [16]. The second group for sub-analysis included residents (0 cases), novice robotic surgeon (0 cases), intermediate (1–49 cases), experienced (50–200) and expert (>200) [17, 18].

### Outcomes

We hypothesized that the overall score on the virtual simulation module “Tubes” without warm up is able to distinguish novice surgeons from intermediate and experienced surgeons. Our primary outcome was the overall score on the advanced suturing virtual simulation “Tubes” module as a continuous variable. A secondary outcome was the difference in the scores between the first and second attempt, which we hypothesized would be more distinct in novice surgeons. We then examined the individual metrics to determine any trends that categorize common mistakes between novice and experienced robotic surgeons.

### Statistical analysis

We investigated the correlation of the number of robotic cases and the individual overall performance MScore on the first attempt. Subsequently, we compared the mean overall MScore on the first attempt comparing novice robotic surgeons (<10 cases) to more experienced robotic surgeons (>10 cases) using the *t*-test. Each sub-group’s (resident, novice, intermediate, and experienced) achievement of overall MScore was compared to the “expert” group scores using the Wilcoxon Rank test with Benjamini and Hochberg adjustment. In order to investigate the amount of improvement from the first attempt to the second, a paired *t*-test was utilized and is displayed using a bar bell graph for each group. Secondarily, we compared each individual component metric that makes up the Mscore from the first and second attempt using the Students *t* test to determine differences in specific areas. We investigated the correlation of the number of robotics cases compared to the overall Mscore on the first and second attempts at the “Tubes” task on the simulator using Spearman's rho. Univariate and multivariate analyses were performed using logistic regression for individual components of the overall score to determine in which areas the novice robotic surgeons perform poorly. Subsequently, we performed bidirectional stepwise multiple logistic regression to find the most significant of these metrics compared to overall score. Finally, we attempted to determine a cut point between 50 % and 80 % that could be used as the MScore percent, which would maintain a difference between novice and more experienced robotic surgeons while maximizing the number of surgeons who would qualify as proficient to use the robot. All p values <0.05 are considered significant. Statistical analysis was performed using the R statistical package.

## Results and discussion

We enrolled 38 subjects in the study with a previous experience range from 0–2,000 robotic cases. All participants completed the “Tubes” module twice, in which raw and percentage values were obtained for the overall score and the 9 individual metrics included raw and software calculated percent (%) score. The median time to complete the task was 4.5 (2.5–13.6) min on the first attempt and 3.9 (1.9–14.2) min on the second attempt. In addition to the overall score, the individual metrics that improved from the first attempt to the second were: use of the workspace, economy of motion, missed targets, and time to complete the task (Table 2).

**Table 2:** MScores

Metrics	N	1st attempt	2nd attempt	*P* value
		Median/Mean (SD)	Median/Mean (SD)	Paired student’s *t* test
Overall MScore	38	47.5/53.76 (22.11)	62.5/62.58 (20.73)	0.012
Workspace	38	9/9.08 (1.88)	8/8.16 (1.26)	0.015
Collisions	38	5.5/8.32 (7.73)	5/7.24 (8.06)	0.474
Economy of Motion	38	478/510.4 (196.7)	385/440.11 (207.38)	0.043
Excessive For	38	0.0/0.68 (1.65)	0.0/ 2.58 (12.81)	0.376
Instruments Out of View	38	0.0/1.52 (2.73)	0.5/1.45 (2.14)	0.865
Missed Targets	38	7/9.08 (7.57)	4/5.71 (6.24)	0.029
Time to complete task	38	272/325.7 (150.6)	236/260.42 (122.66)	0.011

Overall MScores obtained by subjects based on experience group in the first and second attempt to complete the “Tubes” simulator module

**Fig. 2:**
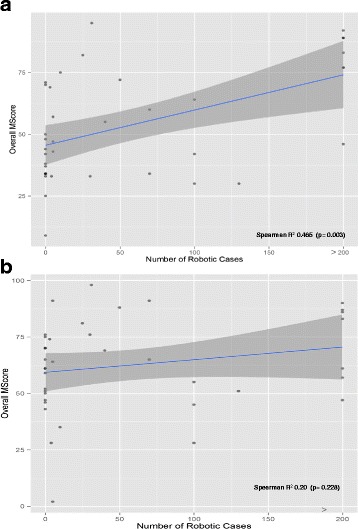
**a** and (**b**): comparison of MScore and number of robotic cases in the first (**a**) and second (**b**) attempt. Scatter plot and regression line comparing the first attempt overall MScore on the Tubes module compared to number of previous robotic cases performed as the attending surgeon. The dark grey shaded area represents a 95 % confidence interval of a linear regression line. The statistical analysis uses the Spearman Rho

**Fig. 3:**
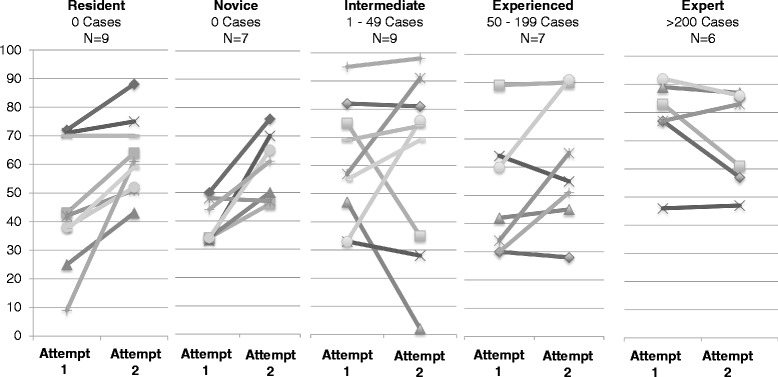
Bar bell plots comparing the first and second attempts on the simulator per subject in each category of experience. The first dot corresponds to the individual subject’s first attempt at the “Tubes” task on the robotic simulator. The second dot to the left is the second attempt for that individual subject. Varying color lines to represent the individual subject’s scores connect the two dots

Educational experience consisted of residents (n = 9), novice robotic surgeons (n = 7), intermediate (n = 9), experienced (n = 7), and expert robotic surgeons (n = 6). Compared to expert surgeons, residents in training and novice robotic surgeons showed significantly lower overall MScores on the Tubes module on both attempts (first attempt p = 0.039 and second attempt p = 0.023) (Table 3). The median overall MScore on the first attempt was 47.5 (range 0–95) and the second attempt was 62.5 (range 2–98). The MScore on the first attempt was correlated to the number of previous robotic cases (Spearman Correlation 0.465 (p = 0.003) (Fig. 2a). The second attempt was no longer correlative (Spearman 0.200; p = 0.228) (Fig. 2b). Significant differences in the mean MScores were noted comparing the novice robotic surgeons and surgeons with some robotic experience on the first (44.7 vs. 63.9; p = 0.005) and second attempt (56.0 vs. 69.9; p = 0.037). The overall scores did improve on the second attempt by 8.82 %, in which the novice group improved to a greater extent than the experienced group (11.4 % vs. 6 %; p = 0.012). We graphed each individual subject’s 1^st^ and 2^nd^ attempts and connected each score with a line to display trends of improvement within each sub-group of experience level (Fig. 3). In this figure, novice and residents without attending level experience on the robot nearly unanimously improved on the second attempt. The other levels of robotic experience seem to be less predictable, possibly due to their particular robotic expertise or experience with this particular simulator.

**Table 3:** Overall MScores based on experience level

Overall MScore								
			Median/Mean overall Mscore (SD)					
Experience level	N	Number of robotic cases	1st attempt	*P* value*	2nd attempt	*P* value*	Mean difference (SD)	*P* value*
Resident	9	0	42/45.22 (21.94)	0.039	61/62.56 (13.70)	0.97	17.33 (14.99)	0.023
Novice	7	0	34/39.57 (7.48)	0.039	61/59.29 (11.86)	0.97	19.71 12.75)	0.023
Intermediate	9	1-49	57/60.67 (21.42)	0.28	74/61.56 (32.35)	0.97	0.89 (29.32)	0.555
Experienced	7	50 - 200	42/49.86 (22.12)	0.149	55/60.71 (23.23)	0.97	10.86 (16.50)	0.305
Expert	6	>200	80/77.33 (16.52)	-	72/70.17 (17.28)	-	-7.17 (11.43)	-
				*P* Value**		*P* Value**		*P* Value**
Novice	20	≤10	42.5/44.65 (16.91)	0.006	60/56.00 (19.83)	0.037	11.4 (23.2)	0.429
Experienced	18	>10	68/63.89 (23.21)		72.5/69.8 (19.70)		5.9 (17.3)	

*Compared to Expert (Wilcoxin Rank with Benjamini and Hochberg adjustment)

**Student *t* test

In order to identify metrics most influential to the overall MScore, we performed logistic regression and identified that experienced surgeons had more out of view penalties if adjusting for missed targets (p = 0.014) on the first attempt despite having higher overall scores. On the second attempt, time (p = 0.014) and missed targets (p = 0.004) were the most significant factors between the novice and experienced groups (Table 4).

**Table 4:** Experience and individual metrics of the overall MScore

Univariate	*Attempt 1*	*Attempt 2*
	*P* value	*P* value
Workspace	0.943	0.586
Collisions	0.136	0.081
Economy of Motion	0.104	0.121
Excessive Force	0.101	0.3
Out of View	0.025	0.546
Missed Targets	0.083	0.91
Overall Time	0.021	0.13
Multivariate^a^		
Excessive Force	0.078	
Out of View	0.014	
Missed Targets	0.127	0.014
Overall Time		0.004

Metrics obtained by subjects based on experience group (novice ≤10 cases, experienced >10 cases) in the first and second attempt to complete the “Tubes” simulator module

^a^Using Akaike Info Criterion Model Selection

Therefore, the “Tubes” simulator module within the DVSSS does have construct validity to determine if the subject has performed more than 10 cases previously. The virtual simulation task, therefore, may be useful as an objective assessment of proficient use of the robotic console defined as basic functional use of the robot system (not surgical proficiency). Limiting the test to only one difficult virtual reality simulation may limit the amount of information obtained, however, the test can be performed quickly (approximately 5 min) and efficiently.

A previous study has suggested that the use of virtual reality robotic simulation may serve as an assessment tool in a variety of settings [19]. We tested a wide range of robotic surgical experience to determine if one task or module (“Tubes”) could have the ability to provide assessment value in proctoring in a future study. Proctoring requires another surgeon to assess the new surgeon’s ability to perform a particular surgery and report to the credentialing authority [20]. The proctor’s prior training, experience and ability to judge competency may be highly variable; however, the proctor does have the authority and responsibility to recommend further training prior to a surgeon being given unrestricted privileges to perform robotic surgery [10, 20]. Therefore, introducing an objective measure to assess the surgeon’s comfort with the robotic system may be helpful to identify those surgeons who may need to take part in a standardized robotic curriculum prior to robotic privileging.

The simulator can identify particular tasks the user may need additional practice or training on, making the test a learning opportunity (Fig. 1). Metrics such as workspace utilization, economy of motion, missed targets and time all play important factors in the overall MScore and can provide an opportunity for self-reflection and improvement. No to be understated, the more experienced group of robotic surgeons had more “out of view” errors. More experienced surgeons may be moving robotic arms out of the field, potentially causing safety concerns to which a common reaction would be: “I know where the arm is.” Even experienced surgeons should use the simulation as an opportunity for improvement. Another scenario would be if a novice scored exceptionally well on the MScore tasks. The surgeon would still go through the usual proctoring and prove the ability to troubleshoot the robotic system, but may not need additional mentored robotic console training.

The simulator can provide these metrics; however, in order to incorporate them into proctoring, a benchmark needs to be set to provide assessment. Many proficiency based surgical curricula and training programs are based on pass rates of 80 %–91 % to progress to the next level [9, 21]. We then identified that an overall MScore of 75 % would serve as the lowest possible score that could still distinguish surgeon experience even if the surgeon is granted a second attempt. On first attempt only 1 of 20 (5 %) novice surgeons were able to pass compared to 8 of 18 (44 %) surgeons with >10 robotic cases (p = 0.001). When given a second attempt, 2 additional novices (15 %) and 1 additional surgeon with some robotic experience (50 %) were able to pass with maintenance of statistically significant difference between the groups (p = 0.020). Overall, 23 failed both attempts and 6 passed both attempts, 3 passed on the first but not second and 6 failed on first and passed on second attempt. We emphasize the contrast in distinguishing familiarity with the robotic console and basic operation, not surgical proficiency. The cutoff values are arbitrary but should be consistent to compare surgeons to their peers and provide baseline proficiency.

If a surgeon “fails” their first attempt, the natural inclination is to try again. Therefore, we had the subjects perform the task a second time to determine improvement levels, as described above. We found that both the novice and experienced groups were able to improve their overall MScore on the second attempt, with the novice group able to improve to a greater extent. The improvement provides suggestive information that the simulator does have a learning curve and repeated measures may improve their virtual reality score, which may in turn provide improved operative efficiency [9]. Thus, turning the proctoring experience into an opportunity for improvement with specific recommendations on areas of focus. Based on the simulator results, the learner can be directed to a surgeon-specific training curriculum if needed.

The culture regarding operative safety has drastically improved in the last few decades with the use of safety checklists, pathways, and guidelines [22]. One component of safety that has not been investigated sufficiently is the incorporation of new technology and the surgeon. Previous studies suggest a reduction in errors may be achieved with the use of surgical simulation similar to flight simulation in the aviation industry [23]. Currently, simulators for surgical training, practice and warm-up are not widely utilized [24]. Barriers of cost, validation, and optimal specific simulators and tasks have hampered widespread adoption.

Our study is limited by the sample size and number of repeated measures. In addition, we relied on the surgeons to remember the number of robotic cases they have performed, which may be subject to recall bias. Of note, we did not ask their previous experience with simulators. The study was developed in the context of a robotic training course, which interjects some bias regarding the participants. The novice surgeons and residents are not yet at the point of requiring robotic surgical privileges, although a range of skill was needed for the purposes of the study. Additionally, the use of surgical simulators have limitations in that the DVSSS is attached to the actual console and can only be performed when not in use for patient care. Similar software has been used in the Da Vinci Trainer (Mimic Technologies, Seattle, WA, USA) as a tabletop simulator that may be more mobile though may have less working space [12]. The simulator’s virtual reality environment is improving but continues to have low fidelity compared to actual human surgery. Therefore, simulator based testing can only offer an assessment regarding operation of the robotic equipment and not surgical decision-making. We stress that this single simulator test is helpful, but may not be ready for widespread use and standardization. Subjects may not be familiar with the simulator and may need to perform practice sessions first; due to time constraints of the study, we selected one tool and performed it twice. The use of simulators prior to incorporating them into credentialing should be rigorously studied and tested, such as the Fundamentals of Laparoscopic Surgery examination for general surgeons [25, 26]. Proctors with robotic experience would be needed to evaluate actual robotic surgical proficiency. With the help of telemedicine, these opportunities may be offered to hospitals that do not have experienced robotic surgeons available [27].

## Conclusions

A single virtual simulator exercise can provide objective information to assist surgical proctors in assessing the use of the surgical robot in addition to the usual proctoring process. This study supports further research regarding the proctoring process for robotic privileges and further incorporation of simulation into robotic skills testing. The results from the virtual simulation process may be used as a learning tool and guideline for individualized robotic curriculum to improve the surgeon’s efficiency on the robotic console.
